# Annual Out-of-Pocket Expenditures and Financial Hardship Among Cancer Survivors Aged 18–64 Years — United States, 2011–2016

**DOI:** 10.15585/mmwr.mm6822a2

**Published:** 2019-06-07

**Authors:** Donatus U. Ekwueme, Jingxuan Zhao, Sun Hee Rim, Janet S. de Moor, Zhiyuan Zheng, Jaya S. Khushalani, Xuesong Han, Erin E. Kent, K. Robin Yabroff

**Affiliations:** ^1^Division of Cancer Prevention and Control, National Center for Chronic Disease Prevention and Health Promotion, CDC; ^2^Surveillance and Health Services Research, American Cancer Society, Atlanta, Georgia; ^3^Division of Cancer Control and Population Sciences, National Cancer Institute, Rockville, Maryland; ^4^ICF International, Fairfax, Virginia.

In the United States in 2019, an estimated 16.9 million persons are living after receiving a cancer diagnosis (*1*). These cancer survivors face many challenges, including functional limitations, serious psychological distress (*2*), and other lasting and late effects of cancer treatments. Because of the high cost of cancer therapy, many cancer survivors are more likely to face substantial out-of-pocket health care expenditures and financial hardship, compared with persons without a history of cancer (*3**,**4*). Out-of-pocket expenditures and financial hardship associated with cancer have been higher among survivors aged 18–64 years than they have been among older survivors (*5*). To estimate annual out-of-pocket expenditures and financial hardship among cancer survivors aged 18–64 years, compared with persons without a cancer history, CDC, the American Cancer Society, and the National Cancer Institute analyzed data from the 2011–2016 Medical Expenditure Panel Survey (MEPS).* The average annual out-of-pocket spending per person was significantly higher among cancer survivors ($1,000; 95% confidence interval [CI] = $886–$1,113) than among persons without a cancer history ($622; CI = $606–$639). Financial hardship was common; 25.3% of cancer survivors reported material hardship (e.g., problems paying medical bills), and 34.3% reported psychological hardship (e.g., worry about medical bills). These findings add to accumulating evidence documenting the financial difficulties of many cancer survivors. Mitigating the negative impact of cancer in the United States will require implementation of strategies aimed at alleviating the disproportionate financial hardship experienced by many survivors. These strategies include systematic screening for financial hardship at cancer diagnosis and throughout cancer care, integration of discussions about the potential for adverse financial consequences of treatments in shared treatment decision-making, and linkage of patients and survivors to available resources to ensure access to high-quality evidence-based care.

MEPS is conducted by the U.S. Department of Health and Human Services’ Agency for Healthcare Research and Quality and is an annual, nationally representative household survey of the civilian noninstitutionalized population that collects detailed information on demographic characteristics, health status, health insurance coverage, household income, and health care expenditures, including out-of-pocket spending. This report used pooled data from the 2011–2016 MEPS (average annual response rate of 46.0%) and the 2011 and 2016 MEPS Experiences with Cancer self-administered questionnaires completed by cancer survivors (response rates of 90.0% and 81.2%, respectively). MEPS self-administered questionnaires included questions about how cancer, its treatment, and lasting effects of treatment affected access to care, employment, and financial situation. All analyses were conducted using SAS (version 9.4; SAS Institute) and Stata/IC (version 14; StataCorp) to account for the complex survey design and nonresponse. Statistical tests were two-sided, and differences were considered statistically significant if p<0.05.

Cancer survivors were identified as persons who responded affirmatively to the MEPS question “Have you ever been told by a doctor or other health professional that you had cancer or a malignancy of any kind?” Out-of-pocket spending was estimated in two ways: 1) annual out-of-pocket spending in 2016 dollars (https://www.bea.gov/) and 2) high annual out-of-pocket burden (defined as spending >20% of annual family income on medical care). Multivariable generalized linear regression with a gamma distribution and a log link was used to estimate annual out-of-pocket spending, comparing persons with and without a cancer history, and adjusted for the following sociodemographic characteristics: age group, sex, race/ethnicity, health insurance status, employment status, number of MEPS priority conditions^†^ (excluding cancer), marital status, and educational attainment. Multivariable logistic regression was used to evaluate the association between cancer history and high annual out-of-pocket burden adjusted for the same sociodemographic characteristics.

Financial hardship associated with cancer, its treatment, or the lasting effects of that treatment was measured in material and psychological domains. Material hardship was measured by asking survivors whether they ever had to borrow money, go into debt, or file for bankruptcy or had been unable to cover their share of medical costs. Psychological hardship was considered being worried about large medical bills. The percentages of material and psychological financial hardship were estimated using multivariable logistic regression analyses adjusted for the same sociodemographic characteristics.

Cancer survivors were more likely to be older, female, non-Hispanic white (white), married, privately insured, working full-time, and have higher education and multiple chronic conditions than were persons without a cancer history (Table 1). Approximately one half of cancer survivors (54.2%) received their diagnosis at least 5 years before the survey. In unadjusted analysis, cancer survivors had higher mean annual out-of-pocket expenditures and were more likely to have high out-of-pocket burden than were persons without a cancer history.

**TABLE 1:** Number and percentage of cancer survivors and persons without a history of cancer, aged 18–64 years (N = 123,771), by demographic characteristics — Medical Expenditure Panel Survey (MEPS), United States, 2011–2016

Characteristic	Cancer survivors* (n = 4,753) % (95% CI)	Persons without a history of cancer* (n = 119,018) % (95% CI)	Chi-square p-value
**Age group at interview (yrs)**			
18–39	15.9 (14.3–17.5)	48.9 (48.1–49.6)	<0.001
40–49	19.6 (17.9–21.4)	20.5 (20.0–21.0)	
50–64	64.6 (62.2–66.8)	30.6 (29.9–31.3)	
**Sex**			
Men	34.5 (32.1–37.0)	49.9 (49.5–50.2)	<0.001
Women	65.5 (63.1–68.0)	50.1 (49.7–50.5)	
**Race/Ethnicity**			
White, non-Hispanic	76.5 (74.4–78.4)	61.2 (59.2–63.1)	<0.001
Black, non-Hispanic	8.7 (7.6–9.9)	12.5 (11.3–13.8)	
All other races/ethnicities	14.9 (13.1–16.8)	26.3 (24.3–28.3)	
**Marital status**			
Married	60.6 (57.8–63.2)	51.4 (50.5–52.3)	<0.001
Not married^†^	39.5 (36.8–42.2)	48.6 (47.7–49.5)	
**Education**			
Less than high school graduate	10.9 (9.6–12.3)	14.1 (13.5–14.8)	<0.001
High school graduate	26.4 (24.2–28.7)	27.5 (26.7–28.3)	
Some college or more	62.7 (60.3–65.1)	58.4 (57.4–59.5)	
**Health insurance**			
Any private	71.9 (69.7–74.1)	71.3 (70.1- 72.5)	<0.001
Public only**^§^**	19.2 (17.2–21.3)	13.1 (12.3–13.9)	
Uninsured	8.9 (7.5–10.5)	15.6 (14.8–16.5)	
**Family income**			
Poor (<100% FPL)	14.4 (13–15.8)	12.9 (14.3–16.1)	0.0604
Near poor and low income (100%–200% FPL)	15.5 (14.1–17.0)	16.5 (17.4–18.6)	
Middle and high income (>200% FPL)	70.1 (68.0–72.2)	70.6 (65.6–68.1)	
**Employment status**			
Full-time	54.2 (51.7–56.6)	64.2 (45.3–46.7)	<0.001
Part-time	4.8 (3.8–6.0)	5.7 (4.0–4.4)	
Not working	41.0 (38.7–43.4)	30.2 (49.2–50.5)	
**MEPS priority conditions** ^¶^			
Zero or one	47.8 (45.3–50.3)	75.4 (74.8–76.0)	<0.001
Two	20.6 (18.8–22.5)	12.8 (12.4–13.1)	
Three or more	31.6 (29.2–34.2)	11.9 (11.4–12.3)	
Yrs since last cancer treatment**			
<5	45.5 (41.8–50.0)	N/A	N/A
≥5 or never treated/Missing	54.2 (50.0–58.2)	N/A	N/A
**Out-of-pocket health care expenditure**			
% with high out-of-pocket burden^††^	2.3 (1.8–2.9)	1.0 (0.9–1.1)	<0.001
Mean (95% CI), $	1,158 (1,051–1,265)	564 (546–583)	<0.001
Median (IRQ), $	488 (1,271)	135 (554)	<0.001

**Abbreviations:** CI = confidence interval; FPL = federal poverty level; IQR = interquartile range; N/A = not applicable.

* Sample sizes were unweighted.

^†^ Not married included widowed, divorced, separated, or never married.

^§^ Public insurance included Medicare, Medicaid, State Children’s Health Insurance Program, and/or other public hospital/physician coverage. TRICARE and CHAMPVA were treated as private coverage, as were employer-based, union-based, and other private insurance.

^¶^ Conditions included arthritis, asthma, diabetes, emphysema, heart disease (angina, coronary heart disease, heart attack, or other heart condition or disease), high cholesterol, hypertension, attention deficit hyperactivity disorder or attention deficit disorder, and stroke, and excluded cancer.

** Years since last cancer treatment top-coded at ≥20 by MEPS. This question was only asked of cancer survivors who participated in MEPS Experiences with Cancer Survey in 2011 or 2016.

^††^ High health care out-of-pocket burden was defined as having annual out-of-pocket expenditures on health care services >20% of annual family income.

In adjusted analyses, mean annual out-of-pocket spending was $1,000 (CI = $886–$1,113) for cancer survivors and $622 (CI = $606–$639) for persons without a cancer history (p<0.001) (Table 2). Cancer survivors also had higher annual out-of-pocket expenditures than did persons without a cancer history in each sociodemographic stratum. Annual out-of-pocket spending was higher among persons with and without a cancer history who were older and who had more MEPS priority conditions.

**TABLE 2:** Mean annual out-of-pocket expenditure and prevalence of high out-of-pocket burden* among cancer survivors and persons without a history of cancer, aged 18–64 years (N = 123,771) — Medical Expenditure Panel Survey (MEPS), United States, 2011–2016

Characteristic	Mean out-of-pocket cost^†^ (2016 U.S. dollars)			High out-of-pocket burden*		
	Cancer survivors (n = 4,753) $ (95% CI)	Persons without a history of cancer (n = 119,018) $ (95% CI)	p-value	Cancer survivors (n = 4,753) % (95% CI)	Persons without a history of cancer (n = 119,018) % (95% CI)	p-value
**Total**	**1,000 (886–1,113)**	**622 (606–639)**	**<0.001**	**1.9 (1.4–2.5)**	**1.0 (1.0–1.1)**	**<0.001**
**Age group at interview (yrs)**						
18–39	907 (722–1,093)	519 (496–542)	<0.001	2.0 (1.1–2.9)	0.8 (0.6–0.9)	<0.001
40–49	1,004 (852–1,156)	586 (557–615)		1.6 (0.7–2.6)	0.9 (0.7–1.1)	
50–64	1,119 (975–1,263)	756 (728–784)		2.0 (1.3–2.8)	1.4 (1.2–1.6)	
**Sex**						
Men	976 (801–1,151)	519 (499–539)	<0.001	2.0 (1.1–2.8)	0.9 (0.8–1.0)	<0.001
Women	1,023 (916–1,129)	721 (697–745)		1.9 (1.4–2.5)	1.1 (1.0–1.2)	
**Race/Ethnicity**						
White, non-Hispanic	1,10 (959–1,244)	715 (693–738)	<0.001	2.2 (1.5–2.9)	1.2 (1.1–1.4)	<0.001
Black, non-Hispanic	639 (517–761)	380 (356–403)		1.0 (0.3–1.7)	0.7 (0.6–0.8)	
All other races/ethnicities	899 (756–1,042)	484 (456–512)		2.0 (1.1–2.9)	0.8 (0.7–0.9)	
**Marital status**						
Married	1,011 (882–1,139)	628 (606–649)	<0.001	1.1 (0.6–1.5)	0.5 (0.5–0.6)	<0.001
Not married^§^	984 (831–1,138)	616 (594–638)		2.8 (2.0–3.5)	1.6 (1.5–1.8)	
**Education**						
Less than high school graduate	731 (566–896)	463 (424–502)	<0.001	1.7 (0.6–2.8)	0.8 (0.7–1.0)	<0.001
High school graduate	914 (707–1,121)	508 (481–535)		1.9 (1.0–2.7)	0.9 (0.8–1.1)	
Some college or more	1,091 (969–1,214)	704 (682–726)		2.1 (1.4–2.7)	1.2 (1.1–1.3)	
**Health insurance status**						
Any private	1,114 (968–1,260)	680 (659–700)	<0.001	1.9 (1.1–2.6)	0.9 (0.8–1.0)	<0.001
Public only^¶^	471 (359–583)	325 (295–355)		1.5 (0.7–2.2)	0.9 (0.7–1.1)	
Uninsured	959 (726–1,193)	647 (604–691)		2.8 (1.4–4.3)	1.9 (1.6–2.2)	
**Employment status**						
Full-time	895 (803–986)	593 (572–613)	<0.001	0.6 (0.3–0.9)	0.5 (0.4–0.6)	<0.001
Part-time	1,057 (780–1,335)	600 (549–651)		2.9 (0.6–5.2)	1.2 (0.8–1.5)	
Not working	1,259 (966–1,552)	697 (664–729)		4.3 (2.9–5.7)	1.9 (1.7–2.2)	
MEPS priority conditions**						
Zero or one	891 (764–1,019)	493 (476–510)	<0.001	1.8 (1.2–2.4)	0.7 (0.6–0.8)	<0.001
Two	1,252 (1,005–1,500)	802 (755–850)		2.6 (1.3–3.9)	1.4 (1.2–1.7)	
Three or more	1,359 (1,174–1,544)	1,138 (1,073–1,203)		2.4 (1.1–3.8)	2.0 (1.7–2.4)	

**Abbreviation:** CI = confidence interval.

* High health care out-of-pocket burden was defined as having annual out-of-pocket expenditures on health care services >20% of annual family income. Predicted high out-of-pocket burden percentages from a logistic model controlling for age, sex, race/ethnicity, health insurance status, employment status, and number of conditions (excluding cancer).

^†^ Predicted mean out-of-pocket costs from a two-part model controlling for age, sex, race/ethnicity, health insurance status, employment status, and number of conditions (excluding cancer). All costs were adjusted to 2016 dollars using the Consumer Price Index for Medical Care.

^§^ Not married included widowed, divorced, separated, or never married.

^¶^ Public insurance included Medicare, Medicaid, State Children’s Health Insurance Program, or other public hospital or physician coverage. TRICARE and CHAMPVA were treated as private coverage, as were employer-based, union-based, and other private insurance.

** Conditions included arthritis, asthma, diabetes, emphysema, heart disease (angina, coronary heart disease, heart attack, other heart condition or disease), high cholesterol, hypertension, attention deficit hyperactivity disorder or attention deficit disorder, and stroke, and excluded cancer.

Cancer survivors were more likely to report high out-of-pocket burden (>20% of annual family income), compared with persons without a cancer history (1.9% versus 1.0%; p<0.001). Among cancer survivors, annual out-of-pocket spending was higher among those with private health insurance coverage than those without health insurance ($1,114 versus $959; p<0.001), but out-of-pocket burden was higher among the uninsured (2.8%) than among those with private insurance (1.9%) or public insurance (1.5%). Out-of-pocket spending was highest among survivors who were not working (4.3%) followed by those who were working part-time (2.9%) and those who were working full-time (0.6%).

In adjusted analyses, approximately one fourth (25.3%) of cancer survivors reported material hardship associated with cancer, and one third (34.3%) reported psychological financial hardship (Table 3). The percentage of survivors who reported experiencing material or psychological financial hardship was higher among minority racial/ethnic groups than among whites and highest for persons aged 40–49 years. Survivors who were uninsured were most likely to report material financial hardship (36.5%) followed by those with public (33.1%) and private (21.9%) insurance. Psychological financial hardship was also higher among the uninsured (49.4%) than among those with public (35.9%) or private (32.5%) health insurance coverage.

**TABLE 3:** Prevalence of material and psychological financial hardship associated with cancer survivors aged 18–64 years (N = 910), cancer treatment, or lasting effects of treatment — Medical Expenditure Panel Survey (MEPS) Experiences with Cancer Survey, United States, 2011 and 2016

Characteristic	Material hardship (need to borrow money, go into debt, declare bankruptcy, or be unable to cover cost share)	Psychological hardship (worry about medical bills)
	% (95% CI)*	% (95% CI)*
**Total**	**25.3 (22.4–28.5)**	**34.3 (30.6–38.1)**
**Age group at interview (yrs)**		
18–39	27.1 (17.8–36.4)	40.5 (29.2–51.8)
40–49	34.2 (26.0–42.4)	47.2 (38.2–56.2)
50–64	22.3 (18.9–25.8)	29.7 (25.4–34.0)
**Sex**		
Men	22.1 (16.7–27.5)	33.7 (26.5–41.0)
Women	26.7 (22.6–30.8)	34.5 (30.2–38.8)
**Race/Ethnicity**		
White, non-Hispanic	23.8 (20.2–27.4)	32.6 (28.0–37.1)
Black, non-Hispanic	31.3 (22.9–39.8)	40.3 (30.9–49.7)
All other races/ethnicities	29.8 (22.4–37.1)	40.7 (32.2–49.1)
**Marital status**		
Married	25.1 (20.4–29.8)	34.7 (30.1–39.3)
Not married^†^	25.6 (20.5–30.6)	33.6 (27.4–39.7)
**Educational attainment**		
Less than high school graduate	27.2 (17.1–37.3)	36.6 (26.5–46.7)
High school graduate	23.6 (18.0–29.2)	32.0 (25.3–38.7)
Some college or more	25.7 (21.9–29.5)	34.8 (30.3–39.4)
**Family income**		
Poor (<100% FPL)	26.8 (17.6–36.0)	30.6 (20.5–40.8)
Near poor and low income (100%–200% FPL)	36.1 (28.5–43.6)	32.8 (24.6–41.1)
Middle and high income (>200% FPL)	22.5 (18.5–26.4)	35.2 (30.3–40.2)
**Health insurance status**		
Any private	21.9 (18.1–25.7)	32.5 (27.9–37.0)
Public only^§^	33.1 (24.1–42.1)	35.9 (26.0–45.7)
Uninsured	36.5 (23.2–49.8)	49.4 (35.4–63.4)
**Employment status**		
Full-time	26.7 (22.0–31.3)	35.0 (30.0–40.0)
Part-time	30.6 (15.4–45.7)	28.7 (10.4–46.9)
Not working	23.0 (17.6–28.5)	34.1 (28.5–39.7)
**MEPS priority conditions** ^¶^		
Zero or one	24.8 (19.7–29.9)	31.0 (25.8–36.1)
Two	22.8 (16.0–29.6)	33.6 (25.2–42.0)
Three or more	27.7 (22.1–33.3)	40.2 (32.9–47.4)
Yrs since last cancer treatment**		
<5	27.8 (22.9–32.6)	40.4 (34.3–46.5)
≥5 or never treated/Missing	23.3 (19.2–27.3)	29.1 (24.3–34.0)

**Abbreviations:** CI = confidence interval; FPL = federal poverty level.

* Predicted percentages from a logistic model controlling for age, sex, race/ethnicity, health insurance status, employment status, and number of conditions (excluding cancer).

^†^ Not married included widowed, divorced, separated, or never married.

^§^ Public insurance included Medicare, Medicaid, State Children’s Health Insurance Program, and/or other public hospital/physician coverage. TRICARE and CHAMPVA were treated as private coverage, as were employer-based, union-based, and other private insurance.

^¶^ Conditions included arthritis, asthma, diabetes, emphysema, heart disease (angina, coronary heart disease, heart attack, other heart condition or disease), high cholesterol, hypertension, attention deficit hyperactivity disorder or attention deficit disorder, and stroke, and excluded cancer.

** Years since last cancer treatment top-coded at ≥20 by MEPS. This question was only asked of cancer survivors who participated in MEPS Experiences with Cancer Survey in 2011 or 2016.

## Discussion

Cancer survivors aged 18–64 years in the United States had higher annual out-of-pocket expenditures and were more likely to report high out-of-pocket burden than were persons without a cancer history. Further, approximately one fourth of cancer survivors reported having material financial hardship, and one third reported having psychological financial hardship associated with cancer, its treatment, or late and lasting effects of treatment. These findings are consistent with other evidence suggesting that cancer survivors experience substantial financial difficulties coping with the costs of health care (*3**,**5**,**6*).

In 2009, the American Society of Clinical Oncology’s Cost of Care Task Force identified the critical role of oncologists in addressing out-of-pocket costs of cancer care with their patients (*7*). Subsequently, in 2013, the National Academies of Science, Engineering, and Medicine (NASEM) described affordable health care as a component of high-quality cancer care.^§^ In 2014, NASEM highlighted the issue of rising cancer drug costs and patient access to affordable and effective drug therapies.^¶^ The 2018 President’s Cancer Panel report, Promoting Value, Affordability, and Innovation in Cancer Drug Treatment, further emphasized the importance of affordability.** These reports and findings from the current study reflect the growing evidence that financial hardship might negatively affect survivors’ health and well-being.

Access to health insurance coverage has been identified as essential to providing affordable cancer care by the American Society of Clinical Oncology (*8*) and NASEM.^††^ Substantial evidence links health insurance coverage with positive health outcomes among cancer survivors (*9*). In this study, uninsured cancer survivors had lower out-of-pocket spending than did survivors with private insurance coverage, but that spending represented a larger proportion of family income. Lack of health insurance coverage was also strongly associated with both material and psychological financial hardship. Even many cancer survivors with private insurance coverage reported borrowing money, being unable to cover their share of medical care costs, going into debt, or filing for bankruptcy.

The findings in this report are subject to at least four limitations. First, self-reported cancer diagnosis was not verified by medical records.^§§^ Second, analyses were not stratified by cancer anatomic site because sample sizes were insufficient; therefore, these data cannot be used by policy makers or providers to determine whether survivors of cancer at certain anatomic sites are more or less likely to experience financial hardship than others. Third, some important clinical characteristics, such as stage of cancer at diagnosis and types of treatment received before the survey, were unavailable in MEPS. Finally, even though comorbidity was included in multivariable models, some out-of-pocket spending in cancer survivors might result from higher comorbidity among cancer survivors. However, measures of material and psychological hardship were specific to cancer, its treatment, and lasting effects of treatment.

This report used the most recent national data available to present evidence of substantial out-of-pocket expenditure, out-of-pocket burden, and financial hardship among cancer survivors aged 18–64 years. The number of Americans with a history of cancer is projected to increase in the next decade, and the economic burden associated with living with a cancer diagnosis will likely increase as well (*10*). The findings in this report might lead to increased awareness in all sectors of the public health and medical community that the rising cost of cancer care is a major barrier to survivors’ well-being. Efforts at the provider, practice, employer, payer, state, and federal levels are needed to develop and implement evidence-based and sustainable interventions (e.g., including systematic screening for financial hardship at cancer diagnosis and throughout the cancer care trajectory, integrating discussions about the potential for adverse financial consequences of treatments in shared treatment decision-making, and linking patients and survivors to available resources) (*4*) to minimize financial hardship for cancer survivors.

**Summary**

**What is already known about this topic?**

Many cancer survivors face substantial economic burden resulting from cancer and its treatment.

**What is added by this report?**

On average, cancer survivors had significantly higher annual out-of-pocket medical expenditures than did persons without a cancer history. Overall, 25% of survivors reported problems paying medical bills, and 33% reported worry about medical bills. Financial hardship was more common among the uninsured than among those with insurance coverage.

**What are the implications for public health practice?**

The population of cancer survivors is growing, and many struggle to pay for medical care. Evidence-based, sustainable strategies by providers, practices, and payers to reduce out-of-pocket costs could be an important component of high-quality cancer care.

## Footnotes

* http://meps.ahrq.gov/mepsweb/.

† https://meps.ahrq.gov/data_stats/MEPS_topics.jsp?topicid=41Z-1.

§ https://www.nap.edu/catalog/18359/delivering-high-quality-cancer-care-charting-a-new-course-for.

¶ https://www.nap.edu/catalog/18956/ensuring-patient-access-to-affordable-cancer-drugs-workshop-summary.

** https://prescancerpanel.cancer.gov/report/drugvalue/pdf/PresCancerPanel_DrugValue_Mar2018.pdf.

†† https://www.nap.edu/catalog/11468/from-cancer-patient-to-cancer-survivor-lost-in-transition.

§§ https://www.air.org/sites/default/files/Health%20Insurance%20Literacy%20brief_Oct%202014_amended.pdf.
